# Reconstruction of Extreme Rainfall Event on September 19-20, 2017, Using a Weather Radar in Bengkulu of Sumatra Island

**DOI:** 10.1155/2020/1639054

**Published:** 2020-07-08

**Authors:** Jaka A. I. Paski, Furqon Alfahmi, Donaldi S. Permana, Erwin E. S. Makmur

**Affiliations:** ^1^Center for Research and Development, Indonesian Agency for Meteorology Climatology and Geophysics, Jakarta 10720, Indonesia; ^2^Marine Meteorological Center, Indonesian Agency for Meteorology Climatology and Geophysics, Jakarta 10720, Indonesia; ^3^Global Atmosphere Watch Station, Indonesian Agency for Meteorology Climatology and Geophysics, Palu 94111, Central Sulawesi, Indonesia

## Abstract

Extreme rainfall accompanied by strong winds hit the province of Bengkulu in the western coastal area of Sumatera Island during September 19-20, 2017, causing floods and landslides in Seluma and Central Bengkulu district. This extreme rainfall was recorded by Bengkulu Meteorological Station about 257.0 mm day^−1^ using rain-gauge observation. The spatial distribution of extreme rainfall cannot be seen if only using a rain-gauge observation in this location. The spatial distribution of extreme rainfall is needed to identify the impact of rainfall on landslides in large areas. The study aims to (1) develop the reconstruction of the spatial distribution of extreme rainfall using weather radar and (2) investigate the trigger that caused extreme rainfall by analyzing the synoptic-scale tropical waves. Each weather radar datum is saved in a Constant Altitude Plan Position Indicator (CAPPI). To get rainfall information, the CAPPI must be derived from Quantitative Precipitation Estimation (QPE) values. In this paper, we derived CAPPI using a Marshall-Palmer reflectivity-rain rate relationship. The result shows that rainfall formed on September 20, 2017, 21.00 UTC with total daily rainfall ranged between 176 and 247 mm in both districts and the mean of total daily rainfall has exceeded the average of monthly rainfall. The analysis of tropical waves suggests that only Kelvin waves were active and served as a possible trigger factor while the Madden-Julian Oscillation (MJO) and Equatorial Rossby (ER) waves were inactive during this extreme rainfall.

## 1. Introduction

On 20 September 2017, an extreme weather event occurred in the province of Bengkulu on the western coast of Sumatra Island causing floods and landslides in several areas such as Kampung Bahari, Bengkulu, Padang Pelasan and Ngalam villages in Seluma district, and Tanjung Raman in Central Bengkulu district. Extreme weather is very likely to occur in the region of Bengkulu Province due to topographic contours and geographical location. The growth of convective clouds is one of the causes of extreme weather in the region which is related to regional and local factors such as tropical cyclone effects, eddy, shear line regions, and coastal shape [1, 2].

During the extreme event, observation in the Bengkulu Meteorological Station recorded the maximum temperature of 26°C while the range of maximum temperature in September is about 29°–32°C. For rainfall measurement, it reached 257.0 mm per day at the Meteorological Station Fatmawati Bengkulu while at Pulau Baai Climatological Station it reached 230.2 mm per day. These values are higher than the monthly average rainfall of September which is only about 220 mm/month for climatology of 1981–2010 so that these are classified as extreme rainfall [3, 4]. The limited number of observations over the Bengkulu region makes extreme rainfall events difficult to be analyzed, and the existence of weather radar data offers a solution for this issue.

C-band weather radar is capable of detecting short-term weather conditions (near real-time) and has a high resolution and wide coverage area up to a radius of 240 km over the Bengkulu area. Weather radar measures electromagnetic radiation from rain clouds and has the potential to estimate rainfall intensity (*R*) by utilizing reflectivity data (*Z*). Weather radar can be used to provide early warning and analysis of extreme weather phenomena such as heavy rain events, tornadoes, gusty winds, and wind shear [5].

This study aims to reconstruct the extreme rainfall event in Bengkulu on 20 September 2017 using weather radar data. Furthermore, the possible trigger of this extreme rainfall was investigated by analyzing the synoptic-scale tropical waves.

## 2. Materials and Methods

### 2.1. Radar Data Processing

**Figure 1:**
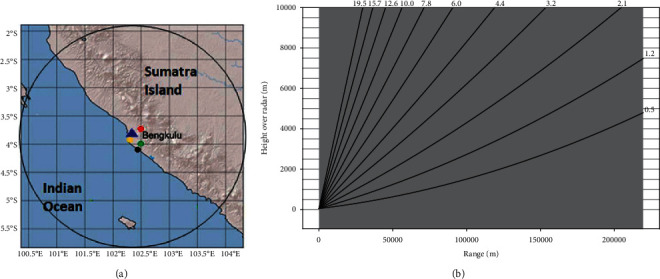
(a) The coverage area of Doppler C-Band weather radar in Bengkulu Province. The red dot is the Tanjung Raman rain gauge, the green dot is the Padang Pelasan rain gauge, the black dot is the Kampung Bahari rain gauge, and the yellow dot is the Desa Ngalam rain gauge. (b) The PPI layers of Bengkulu weather radar.

In this study, the reconstruction of rainfall extreme uses C-band Doppler radar data at Bengkulu Meteorological Station (102.341280°E, 3.858720°S, 45.0 meters above ground level) with Gematronik type and maximum radius coverage of 240 km (Figure 1(a)). Data are recorded every 10 minutes in a volumetric format consisting of 10 Position Plans Indicator (PPI) scans (0.5°, 1.2°, 2.1°, 3.2°, 4.4°, 6.0°, 7.8°, 10.0°, 12.6°, 15.7°, and 19.5°) and each contains reflectivity decibels (dB*Z* with dB*Z* = 10 log *Z*) (Figure 1(b)). For comparison with rainfall station data, four stations are chosen where the incident of floods and the landslide occurred, i.e., in Kampung Bahari (102.309645°E; 3.914793°S), Padang Pelasan (102.447864°E; 4.001139°S), Ngalam Village (102.398262°E; 4.053656°S), and Tanjung Raman (102.503235°E; 3.744831°S).

A python-based open-source software Wradlib was utilized to process weather radar data. Wradlib has been widely used in the processing of weather radar data and its application [6, 7]. It has an important function in weather radar data processing, particularly in generating the Quantitative Precipitation Estimation (QPE) products. An example of its use is for the estimation of rainfall from radar data that shows good results in river flow estimation and simulation of flood events in the Philippines [8] and Bangka Island [9]. Wradlib has been used as a component to develop the Indonesia In-House Radar Integration System (InaRAISE) of BMKG [10, 11].

The reconstruction of extreme rainfall event from 19 to 20 September 2017 utilized all of radar PPI layers and was performed in four stages:

1. Reading the data format, defining the coordinates on the map: Wradlib can be used to process weather radar data of volumetric formats from Rainbow Gematronik. The volumetric data from the radar was then processed and saved into a NetCDF format in Cartesian coordinates. This python programming library was developed by Postdam University and University of Stuttgart.
2. Removal of clutter caused by nonmeteorological factors such as the presence of objects on the surface of the earth (mountains, hills, and tall buildings) or objects in the air (aircraft, birds, etc.) by clutter filters developed by Gabella and Notarpietro [12]. After that, attenuation correction commonly caused by radome (radar cover) is corrected using a method developed by Kraemer and Verworn [13].
3. Gridding: The reflectivity data are displayed on each vertical slope angle of the radar automatically to calculate the value of Constant Altitude PPI (CAPPI), i.e., the horizontal radar reflectivity display at a certain fixed height, and also the maximum CAPPI (CAPPIMAX) value in altitude column. The CAPPI calculation specification has been designed to have a horizontal resolution of 0.5 km/pixel and a vertical resolution of 0.5 km from 0.5 to 10 km height column.
4. Conversion of reflectivity (dB*Z*) into rainfall intensity (mm/hr): estimated rainfall or QPE was derived from a common *Z*-*R* relationship of Marshall-Palmer with *Z* = 200R1.6 for general precipitation [11].

### 2.2. Tropical Wave Analysis

Tropical wave analysis was conducted by investigating the intraseasonal synoptic-scale variation of atmospheric conditions during the event over Bengkulu Province. The investigated tropical waves include the Madden-Julian Oscillation (MJO), Equatorial Rossby (ER) wave, and Kelvin waves.

## 3. Results and Discussion

### 3.1. Event of Reconstruction

**Figure 2:**
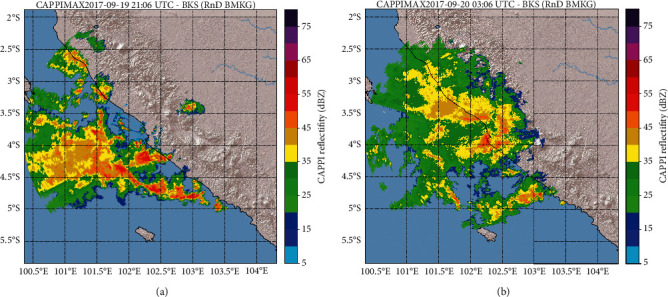
The maximum of the CAPPI reflectivity of Bengkulu Radar. (a) CAPPIMAX represents that the MCS was located in the Indian Ocean on 19^th^ September 2017 at 21 : 06 UTC (20^th^ September 2017 at 04.06 LT) and (b) the MCS was located in Bengkulu Province on 20^th^ September 2017 at 03 : 06 UTC (20^th^ September 2017 at 10 : 06 LT).

Cumulonimbus clouds that caused extreme weather in the Bengkulu area were detected developing over the ocean. Rain clouds were moving from the west (Indian Ocean) to the east (Sumatra Islan) as shown in Figures 2(a) and 2(b). Based on the shape, extent, and life period, these rain clouds include the Mesoscale Convective System (MCS) phenomenon. The MCS phenomenon produces large clouds of Cumulonimbus (hundreds to thousands of km) with longer lifespans (more than three hours). At the time of this extreme rainfall, Cumulonimbus clouds reach more than 100 km with a lifespan of up to 18 hours in the area around Bengkulu.

Figure 2(a) shows Cumulonimbus clouds associated with the MCS located in the western part of Bengkulu (Indian Ocean). The maximum reflectivity value on 19^th^ September 2017 at 21 : 06 UTC (20^th^ September 2017 at 04.06 LT) is estimated to be 60–65 dB*Z*. This range of reflectivity values indicates the possibility of precipitation in the form of heavy rainfall. The greater the reflectivity value, the greater the intensity of the rainfall. Figure 2(a) showed some of the convective nucleus (Cumulonimbus cloud) stretching on the southwest coast of Bengkulu; the convective nucleus is characterized by high reflectivity values. A Cumulonimbus cloud is also capable of causing strong winds and the downburst process from cloud to surface so that the wave height increases in the Indian Ocean at West Bengkulu.

In Figure 2(b) on 20^th^ September 2017 at 03 : 06 UTC (20^th^ September 2017 at 10 : 06 LT), MCS is already in the Bengkulu region and covers the central area of Bengkulu to the north. The visible reflectivity value is not as large as the MCS in the water area, but the distributed reflectivity value distribution is between 35 and 55 dB*Z* in the land area. There is an interval of about 6 hours for MCS coming from moving waters covering the area of Bengkulu Province. The closure of long-term MCS in the region resulted in continuous rainfall that caused flooding in areas such as Kampung Bahari Bengkulu, Padang Pelasan and Ngalam Village in Seluma District, and Tanjung Raman in Central Bengkulu Regency. Reconstruction of total rainfall that hit the Bengkulu region was done by converting the reflectivity (dBZ) of CAPPIMAX product into rainfall intensity (mm/hr). Estimated rainfall rates are derived from two general Marshall-Palmer (MP) *Z*-*R* relationships with *Z* = 200R1.6 for general precipitation. The accumulated rainfall is calculated for one day from 19 September 2017 at 12 : 00 UTC to 20 September 2017 at 12 : 00 UTC. The results show the entire area of Bengkulu rain with varying intensity, where the highest total rainfall occurred in the waters of the Indian Ocean west of Bengkulu to reach 390 mm/day which is 20–30 km from Bengkulu.

**Figure 3:**
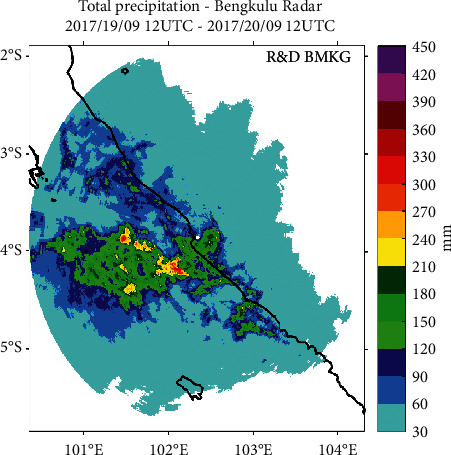
Estimated rainfall during extreme weather events in the Bengkulu region. The value is obtained from the maximum conversion reflectivity of Constant Altitude PPI (CAPPI) products up to a height of 10 km on September 19, 2017, at 12 : 00 UTC to 20 September 2017 at 12 : 00 UTC.

Figure 3 shows, for the area of Bengkulu Province, centralized rain in the city of Bengkulu and surrounding areas such as Seluma and Central Bengkulu. Rainfall reached 270 mm/day for some areas in Bengkulu. There is a white circle on the radar image caused by the Cone of Silence region (a region not detected by radar imagery pans). Cone of Silence is located at the center of the radar due to the radar data emission that forms an angle that is not perpendicular to a radius of up to 3 km. The rain hit throughout the Bengkulu region with a total rainfall of at least 30 mm per day. In the northern coastal area of Bengkulu, the total rainfall reaches 60–90 mm per day. As for the central coastal area to the south of Bengkulu, the total rainfall reaches 120–210 mm per day.

Accumulated rainfall simulation results show that the phenomenon of MCS that caused the occurrence of extreme rain accompanied by strong winds hit the entire west coast of Bengkulu and concentrated in the central Bengkulu region, while for the highest rainfall intensity it is in the waters not far from downtown Bengkulu. QPE simulation also produces rainfall that can describe the situation on the occurrence of floods and landslides in the area of Bengkulu, where there is no weather observation near the area.

At the scene of a flood and landslide in Bengkulu region, simulation of rainfall accumulation resulted from the conversion of radar reflectivity of CAPPIMAX product into QPE value. Simulation of temporal QPE value at the location of the flood shows the rain began to fall at 21 : 00 UTC on September 19, 2017, with a daily accumulation of 236 mm per day in Kampung Bahari Kota Bengkulu, 247 mm per day in Padang Pelasan, 217 mm per day at Ngalam Village Seluma District, and 176 mm per day in Tanjung Raman, Central Bengkulu Regency.

**Figure 4:**
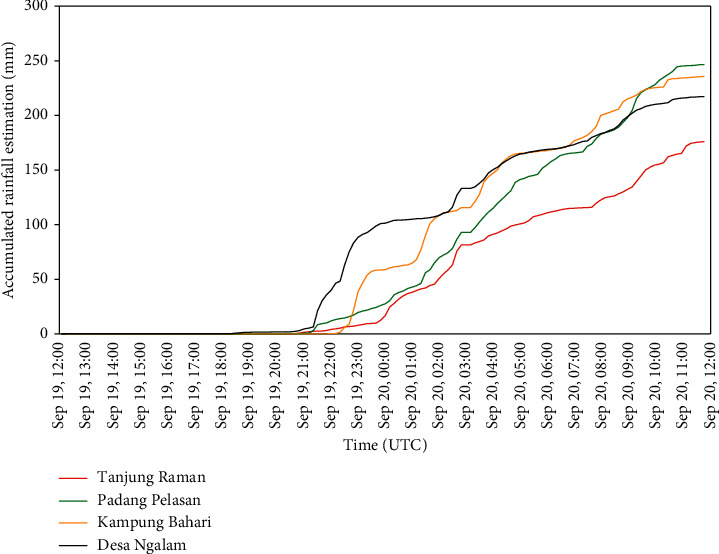
Estimated rainfall during extreme weather events in the Bengkulu region. The value was obtained from Constant Altitude PPI (CAPPI) maximum reflectivity conversion to a height of 10 km on September 19, 2017, at 12 : 00 UTC to 20 September 2017 at 12 : 00 UTC.

From the graph in Figure 4, the village of Ngalam and Padang Pelasan in Seluma District was first washed down by the subsequent rain of the Kampung Bahari area in Bengkulu and Tanjung Raman in Central Bengkulu Regency. At Ngalam Village location within a 2-hour time span from 21 : 00 UTC to 23 : 00 UTC on September 19, 2017, accumulation of rainfall reaches 100 mm with intensity reaching approximately 50 mm per hour. Similarly, the intensity of rainfall in Kampung Bahari at 23 : 00 UTC to 00 UTC reached more than 50 mm per hour. As for the Padang Pelasan and Tanjung Raman areas, the average rainfall intensity is less than 25 mm per hour but rain falls continuously.

### 3.2. Trigger Factor

**Figure 5:**
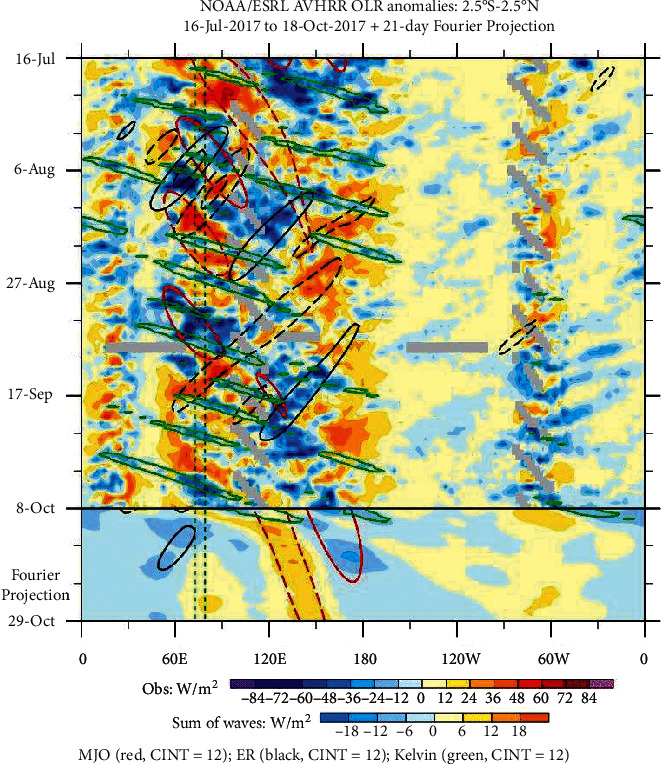
Hovmöllers diagrams of OLR anomalies for monitoring the tropical waves (MJO, ER, and Kelvin) in latitudinal averaging of 2.5 S-2.5 N (https://ncics.org/pub/mjo/archive/2017/2017-10-10/v1/hov/avhrr.waves.NEQ.gif).

On 19-20 September 2017, heavy rainfall triggered floods and landslides on the Indonesian island of Sumatra, particularly in Bengkulu Province (3.8°S; 102.26°E). The synoptic-scale climate variability was analyzed to determine the possible trigger factor, particularly the intraseasonal tropical waves such as Madden-Julian Oscillation (MJO), Equatorial Rossby Wave (ER), and Convectively Coupled Kelvin Wave (CCKW). During this period, the MJO was not active over the eastern Indian Ocean and Sumatra Island (red encircled in Figure 5). During an inactive or weak MJO, Bengkulu usually experiences less rainfall. In this case, MJO is not likely the main trigger for this extreme event. The Equatorial Rossby wave was also not active over Bengkulu (black encircled in Figure 5) and did not contribute to the heavy rainfall event.

On shorter timescales, there was an active CCKW (Kelvin Wave) moving eastward faster than MJO and passed through the Bengkulu and the south Sumatra Island on 19-20 September 2017 (green encircled in Figure 5). This Kelvin wave had a very short period of about a few days over Bengkulu and enhanced the convection and generating MCSs. Therefore, the Kelvin wave is likely the main factor triggered and contributed to the heavy rainfall over the Bengkulu during the period.

## 4. Conclusion

Limitations of surface rain intensity measuring devices led to measurement and analysis of extreme precipitation events causing floods and landslides in Bengkulu using QPE from C-Band Doppler weather radar. The results of atmospheric dynamic reconstruction on September 19-20, 2017, using CMAX radar products show that the MCS moving from the ocean to the mainland with the instability reaches 65 dB*Z*. QPE derived from Marsh-Palmer (MP) ZR relations calculated for one day from 19 September 2017 at 12 : 00 UTC to 20 September 2017 at 12 : 00 UTC shows the entire area of Bengkulu rain with varying intensity, where the highest total rainfall occurred in the waters of the western Indian Ocean Bengkulu to reach 390 mm per day which is 20–30 km from Bengkulu. Temporary QPE values using weather radar have been able to reconstruct the occurrence of extreme rainfall in the area of flood and landslide locations with daily rainfall accumulation of 236 mm per day in Kampung Bahari, 247 mm per day in Padang Pelasan, 217 mm per day in the Ngalam Village, and 176 mm per day in Tanjung Raman. The analysis of tropical waves suggests that only Kelvin waves were active and serve as a possible trigger factor, while the MJO and ER waves were inactive during the event over Bengkulu.

## Data Availability

The data supporting this article is provided within the article. The datasets generated and analyzed during the current study can be obtained from the corresponding author upon reasonable request.
